# Assessment of the strength of recommendation and quality of evidence: GRADE checklist. A descriptive study

**DOI:** 10.1590/1516-3180.2022.0043.R1.07042022

**Published:** 2022-09-12

**Authors:** Camila Torres Bezerra, Antonio José Grande, Vivianny Kelly Galvão, Douglas Henrique Marin dos Santos, Álvaro Nagib Atallah, Valter Silva

**Affiliations:** I PhD. Lawyer, Postgraduate Program on Society, Technologies and Public Policies, Centro Universitário Tiradentes (UNIT), Maceió (AL), Brazil.; II PhD. Adjunct Professor, Postgraduate Program on Infectious and Parasitic Diseases, Faculty of Medicine, Universidade Federal de Mato Grosso do Sul (UFMS), Campo Grande (MS), Brazil; III PhD. Professor, Postgraduate Program on Society, Technologies and Public Policies, Centro Universitário Tiradentes (UNIT), Maceió (AL), Brazil.; IV PhD. Federal Prosecutor and Coordinator, Suboffice of Legal Affairs of the Presidency of the Republic, Brasília (DF), Brazil; V PhD. Full Professor and Head, Evidence-Based Health Department, Universidade Federal de São Paulo (UNIFESP), São Paulo (SP), Brazil; VI PhD. Professor, Postgraduate Program on Society, Technologies and Public Policies, Centro Universitário Tiradentes (UNIT), Maceió (AL), Brazil.

**Keywords:** Evidence-based clinical practice, Systematic review as topic, GRADE approach, Science, Evidence-based medicine, GRADE, Translation of knowledge, Health research evaluation

## Abstract

**BACKGROUND:**

Grading of Recommendations Assessment, Development and Evaluation (GRADE) is a tool for assessing evidence produced in synthesis reports.

**OBJECTIVES:**

To present the translation into Portuguese of the GRADE checklist, whose original version is in English, and to describe and explain each topic, in order to provide examples to researchers and professionals who will use the tool.

**DESIGN AND SETTING:**

Descriptive study developed at Centro Universitário Tiradentes, Maceió, Alagoas, Brazil.

**METHODS:**

This was a translation of the GRADE checklist, with the addition of the Risk Of Bias In Systematic Reviews (ROBIS) tool in the checklist, with examples of its use.

**RESULTS:**

Situations of practical use of the tool were presented in order to facilitate and expand the use of assessment of the quality and strength of evidence among Portuguese speakers.

**CONCLUSIONS:**

The GRADE checklist is valuable in helping to assess the strength and quality of evidence for synthesis reports for healthcare decision-making.

## INTRODUCTION

Grading of Recommendations Assessment, Development and Evaluation (GRADE) is a tool for evidence quality assessment and strength-of-evidence recommendation. This system was developed to provide transparency and to structure the evidence synthesis development process, such as through guidelines, systematic reviews, economic analysis, etc.^1^

According to the tool, the process of evaluating the quality of evidence is separate from the process of formulating recommendations. In the case of a systematic review, the quality of the evidence will reflect the confidence that the effect estimates are correct. Regarding the strength of the recommendation, the quality reflects the confidence that the effect estimates are adequate for supporting a specific recommendation, thus helping the decision-making process.^2^

Thus, when using GRADE approach in a synthesis of the quality of evidence from experimental and observational studies, the levels of evidence are classified (for each outcome studied) on a four-level scale: very low, low, moderate or high.^3^

As established by the tool, the set of available evidence from randomized clinical trials has an initial quality-of-evidence rating of “high”, given that these are experimental studies. If the evidence comes from observational studies, it start with a low quality-of-evidence level.^3^

Furthermore, the quality of evidence can become compromised by factors within five domains, with the consequence of lowering its initial classification. These domains are the following: risk of bias, imprecision, inconsistency, indirect evidence of study results and publication bias. Particularly for observational studies, the GRADE system establishes three criteria that can raise the level of evidence: large magnitude of effect, residual effect of confounding variables and dose-response gradient.^2^

From a practical perspective, correct use of the tool allows healthcare decision-making to be informed by the best evidence, with a lower degree of uncertainty and, consequently, less likelihood of error for patients, the healthcare system and decision-makers.

From a theoretical perspective and to improve the use of the tool, a checklist of questions was developed in 2014 to guide researchers and healthcare professionals in extracting the information that is necessary to conduct an evaluation: a GRADE checklist.^4^

For people who have never done a systematic review, or who have no experience in critically evaluating scientific literature, using the GRADE tool can be a challenge. Professionals who are in healthcare services making decisions on a daily basis need mechanisms that contribute to their understanding of how the evidence was evaluated, or if necessary, need to learn how to evaluate the evidence studied.^5^

The GRADE checklist is an important tool for researchers and other professionals that helps them in the process of evaluating the quality and strength of evidence through a systematic approach. This is because it provides a structure of questions for each of the five domains responsible for downgrading the evidence, which helps raters to detail each item critically and reproducibly, for analysis through GRADE.^4^

## OBJECTIVE

The purpose of this article was to present the translation of the GRADE checklist and to describe and explain each topic, with the aim of reaching out to researchers and professionals who will use the tool.

## METHODS

Given the need to facilitate and expand the use of the GRADE system, a checklist of questions was developed in 2014 to guide researchers and healthcare professionals in extracting the information needed to conduct an assessment using GRADE.^4^

The aim of this checklist was to assist in enhancing the reproducibility of GRADE assessments, given that this system can present a certain degree of complexity. Use of this checklist would be very helpful for assessors with regard to transparently identifying and extracting information that is needed. Through this approach, the items used are clearly identified, in a perfectly reproducible manner, which allows repetition to confirm the results found.^4^

Two medical students translated the checklist into Portuguese under the supervision of a senior researcher.

## RESULTS

A free translation is presented in Table 1, along with analysis of methodological quality and the degree of evidence for systematic reviews of randomized clinical trials.

**Table 1.** Meader checklist for assessing methodological quality and the degree of evidence for randomized clinical trials^4^

**Study limitations (risk of bias)** ^a^	Was random sequence generation used (i.e. no potential for selection bias)? Was allocation concealment used (i.e. no potential for selection bias)? Was there blinding of participants and practitioners (i.e. no potential for performance bias)? Was there blinding of the outcome assessment (i.e. no potential for detection bias)? Were objective outcomes used? Were more than 80% of the participants enrolled in the trial included in the analysis (i.e. no potential reporting bias)? Was the data reported consistently for the outcome of interest (i.e. no potential selective reporting)? Did the tests finish as scheduled (i.e. they did not stop early)? Was random sequence generation used (i.e. no potential for selection bias)?
**Inconsistency** ^b^	Does the estimated point not vary widely? How much do the confidence intervals overlap? All: confidence intervals overlap in at least one of the estimated points of the studies included; Some: confidence intervals overlap, but not all overlap, at least at one of the estimated points; None: There is at least one outlier; the confidence intervals of several studies included do not overlap at most of the estimated points. Is the direction of effect consistent? What is the magnitude of the heterogeneity estimated through the I² statistic? Low: I² < 40%; Moderate: I² = 40%-60%; High: I² > 60%. Was the test for heterogeneity not statistically significant (P > 0.1)?
**Indirect evidence** ^c^	Does the population included in the study have applicability in the context of decision-making? Do the interventions in the studies included have applicability in the context of decision-making? Is the outcome included not a surrogate outcome? Was the outcome assessment time sufficient? Were the conclusions based on direct comparisons?
**Imprecision** ^d^	Is the confidence interval for the pooled estimate (meta-analysis) consistent with benefit or risk? What is the magnitude of the median sample size (high: > 300 participants; intermediate: 100-300 participants; low: < 100 participants)? What is the magnitude of the number of studies included (large: &gt; 10 studies; moderate: 5-10 studies; small: < 5 studies)?^e^ Is the outcome a common event (e.g. more than 1/100 occurrences)?
**Publication bias** ^e^	Was a broad search performed? Was gray literature sought after? Were there no restrictions on study selection based on language? Is there no industry influence on the studies included in the review? Is there evidence of asymmetry in the funnel plot? Are there no discrepancies between the published and unpublished findings of the studies?

^a^Risk-of-bias questions are answered in relation to most evidence pooled in the meta-analysis, rather than in individual trials

^b^Questions about inconsistency are mainly based on visual assessment of forest plots and on statistical quantification of heterogeneity based on I²

^c^In judging the width of the confidence interval, it is recommended that a clinical decision threshold is used to assess whether the imprecision is clinically significant

^d^The questions address search strategy breadth, industry influence, funnel plot asymmetry and discrepancies between published and unpublished studies

^e^Depends on the context of the systematic review area; N/A = not applicable. Judgments: 1 = yes; 0 = uncertain; -1 = no; NA1 = not evaluated; NA2 = not applicable.

**Box 1** ^2–4,6–13^ presents the factors responsible for lowering the quality of the evidence, together with the three factors responsible for upgrading the quality of the evidence, which are particularly applicable to observational studies.

**Box 1. Explanation for using each domain of the Grading of Recommendations Assessment, Development and Evaluation (GRADE) approach**

**A – Study limitations**

Among the factors that can reduce the quality of evidence pointed out by GRADE, there are study limitations or risk-of-study bias. Bias can be defined as a systematic error, or deviation from the truth, in the results.^7^ Assessing the risk of bias of the studies included in the systematic review is important because it is a way of verifying whether the effects of the analyzed interventions were overestimated or even underestimated.^2^

It is, therefore, an evaluation of the methodological quality of the primary studies analyzed in the systematic review, verifying possible limitations to their designs or executions that, through influencing the estimates of the treatment effect, can lead to inadequate conclusions, thereby affecting the estimate of the effect (systematic reviews) and the recommendation to follow (strength of recommendation). Thus, based on the analysis of these studies, the more serious the limitations are, the more the level of evidence may be lowered.^3^

Today, there are already several tools available to researchers that allow them to assess the risk of bias in scientific research. However, the Guidelines for the Systematic Review of Randomized Clinical Trials^6^ recommend to researchers that the risk-of-bias assessment should be carried out using the new version of the Cochrane risk-of-bias tool (RoB 2),^8^ which is composed of a fixed set of five domains, with a series of “signaling questions” that focus on different aspects of the study design, conduct and reporting, in order to obtain the main information for the analysis of the risk of bias. It is, therefore, a very useful tool for assessing methodological quality.

It is also important to note that biases can have a different impact on each outcome. For example, suppose that, in evaluating the effect of a given surgery among patients with chronic kidney problems, a systematic review identified four eligible studies. All were randomized clinical trials, but without adequate blinding of outcome assessors, thus showing a risk of bias due to lack of allocation concealment. However, this risk of bias can vary between the results, depending on the outcome analyzed: the absence of blinding certainly impacts on outcomes such as quality of life, as this is more prone to subjectivity, so it is necessary to downgrade the level of evidence in this case. But if the outcome is overall mortality, masking can already be considered irrelevant, as the outcome of death does not depend on whether the researcher knows which patient underwent the intervention or not, and therefore is not a reason to downgrade the level of evidence for this outcome. All this needs to be taken into account by the evaluator, to ensure correct judgment of this domain.^9^

The questions about the domain of limitations of the checklist study were prepared based on the items of the Cochrane risk-of-bias tool, in order to establish the main points of analysis for the domain, thus facilitating the subsequent analysis in the GRADE system.^4^ Hence, the following points of analysis need to be answered for each outcome:

1. Was random sequence generation used (i.e. no potential for selection bias)?
2. Was allocation concealment used (i.e. no potential for selection bias)?
3. Was there blinding of participants and practitioners (i.e. no potential for performance bias)?
4. Was the outcome evaluation blinded (i.e. no potential for detection bias)?
5. Were objective outcomes used?
6. Were more than 80% of the participants enrolled in trials included in the analysis (i.e. no potential reporting bias)?
7. Was data reported consistently for the outcome of interest (i.e. no potential selective reporting)?
8. Did tests end as scheduled (i.e. they did not stop early)?
9. Was random sequence generation used (i.e. no potential for selection bias)?

The answers to these questions should vary between the following: 1 = yes; 0 = uncertain; -1 = no; NA1 = not evaluated; and NA2 = not applicable. The questions should be answered taking into account most of the evidence aggregated in the meta-analysis, and not the individual trials. Thus, by carefully analyzing each of these points in the available body of evidence, it will be possible to make a more accurate judgment about the risk of bias of the GRADE tool.^4^

**B - Inconsistency**

According to the GRADE system, the criteria for judging the extent of heterogeneity are based on the similarity of point estimates, the extent of overlapping confidence intervals and statistical criteria (including tests of heterogeneity and I²). If the study methods applied provide a convincing explanation for differences in results between studies, the evaluator can maintain the assessment of the quality of the evidence. However, when the variability in the magnitude of the effect is large and remains unexplained, it is appropriate to downgrade the quality of the evidence due to inconsistency. A strong indication of heterogeneity in a group of randomized clinical trials analyzed in a systematic review, is presented when, for example, some of these studies point to a substantial benefit from a particular drug for a certain health problem, while others do not point to any effect. or damage; rather than finding large versus small effects.^10^

Importantly, for GRADE, if the effect size differs between studies, the inconsistencies may be due to differences in four possible variables: populations (for example, a drug may have greater relative effects in populations with health problems that are treated using this drug); interventions (e.g. a drug may have better effects at higher doses); results (e.g. the effectiveness of a treatment with a particular drug may be better with a longer duration of treatment); and study methods (e.g. experimental studies with higher and lower risk of bias).^3^

Hence, when heterogeneity exists and the evaluator does not attribute the difference to any of these four variables, thus making it difficult to interpret the results such that they cannot be explained, the quality of the evidence is affected. In such cases, therefore, the level of evidence should be downgraded because of inconsistency, by one or even two levels.^2^

In this manner, in short, inconsistency consists of an unexplained heterogeneity of results and gives rise to downgrading of the level of evidence by one or two notches, depending on the magnitude of the inconsistency in the results. In the checklist, the analysis on inconsistency takes place through questions based mainly on visual evaluation of forest plots and on statistical quantification of heterogeneity based on I² and Q statistics, following the directions given by GRADE, including evaluation of subgroups that can explain the inconsistency.^4^

The tool therefore proposes a reflection on clinical and methodological differences between studies in which the outcome was analyzed, through presenting four questions to help in this evaluation, which can have any of the following answers: 1 = yes; 0 = uncertain; -1 = no; NA1 = not evaluated; or NA2 = not applicable:^4^

1. Does the estimated point not vary widely?
2. How much do the confidence intervals overlap?
  All: confidence intervals overlap in at least one of the estimated points of the studies included;
  Some: confidence intervals overlap, but not all overlap, at least at one of the estimated points;
  None: There is at least one outlier; the confidence intervals of several studies included do not overlap at most of the estimated points.

1. Is the direction of effect consistent?
2. What is the magnitude of the heterogeneity estimated through the I^2^ statistic?
  Low: I^2^ < 40%;
  Moderate: I^2^ = 40%-60%;
  High: I^2^ > 60%.”
3. Was the test for heterogeneity not statistically significant (P > 0.1)?

**C – Indirect evidence**

It is a fact that confidence in the results of a study that presents direct evidence is greater. Evidence is direct when a study directly compares interventions of interest to the researchers that are delivered to the populations of interest and in which results important to the researchers and patients are measured.^3^

However, this relationship will not always be available. Thus, the quality of the evidence may decrease when this occurs, given that in these cases it is necessary to resort to indirect evidence. In such cases, inferences are made about the relative effects of two interventions: not based on comparison between them, but rather, through comparison of a third control condition.^11^

Therefore, in analyses on indirect evidence, the evaluator is faced with the lack of a direct answer to the question addressed in the studies available, due to four possibilities: differences in populations, interventions, comparators or outcomes, taking into account the acronym PICO that was previously explained.^2^

As an example, imagine that a systematic review aimed to analyze the effect of a drug for treating morbid obesity, and that studies in which this evaluation was carried out in groups of people in countries in the Americas were selected. It would not be expected that the effect of this drug in European countries would be different, and therefore it would not be necessary to downgrade the level of evidence. However, if these studies analyzed the effectiveness of this drug in adults, generalizing these results to children would constitute a situation of indirect evidence, which would therefore generate less confidence in these results. For this reason, in this case, the level of evidence would need to be downgraded because of the use of indirect population-based evidence.^6^

In the checklist, to analyze indirect evidence, questions about population applicability, interventions, comparators and outcomes are included. In short, in this domain, it is necessary to analyze whether the study met its objectives, i.e. whether what it set out to research was found, or whether there was any deviation of interest in relation to the PICO research question. Five questions guide the classification of evidence in this domain:^4^

1. Does the population included in the study have applicability in the context of decision-making?
2. Do the interventions in the studies included have applicability in the context of decision-making?
3. Is the outcome included not a surrogate outcome?
4. Was the outcome assessment time sufficient?
5. Were the conclusions based on direct comparisons?

As in the previous items, the answers to these questions can vary between the following: 1 = yes; 0 = uncertain; -1 = no; NA1 = not evaluated; or NA2 = not applicable. Thus, this analysis will make it possible to arrive at the best possible judgment of the indirect evidence, in the GRADE tool.^4^

**D - Imprecision**

The GRADE system considers that the 95% confidence interval is the main criterion for decisions about imprecision around differences in effect between the intervention and the control for each outcome analyzed. The confidence interval reflects the impact of random error on the quality of evidence. However, it is important to point out that there are limitations to this reliability, so that even if the confidence interval seems satisfactory, when the effects reported in the study are large and the sample size or the number of events are modest, the evaluator must downgrade the quality of evidence because of imprecision.^12^

Thus, for example, when a researcher seeks to assess the effectiveness of a treatment, but the study includes relatively few patients and few events, with a wide confidence interval, the quality of the evidence must be downgraded due to the imprecision of the results found.^2^

In accordance with the GRADE predictions, imprecision was addressed in the checklist through items relating to the width of the confidence interval, the sample size, the magnitude of the effect between the studies under analysis and whether the outcome occurs commonly. Four questions help in reflecting on this domain, and should be answered as follows: 1 = yes; 0 = uncertain; -1 = no; NA1 = not evaluated; or NA2 = not applicable. These questions are the following:^4^

1. Is the confidence interval for the pooled estimate (meta-analysis) not consistent with benefit or risk?
2. What is the magnitude of the median sample size?
3. What is the magnitude of the number of studies included (large: > 10 studies; moderate: 5-10 studies; small: < 5 studies)?
4. Is the outcome a common event (e.g. occurs in more than 1/100)?

**E-Publications bias**

Publication bias occurs when a reported effect is underestimated or overestimated due to selective publication of studies. Thus, the risk of publication bias may be greater for systematic reviews of observational studies than for reviews of randomized controlled trials.^3^

From this perspective, publication bias occurs when entire studies are not reported. For example, systematic reviews that fail to identify unpublished studies, consisting of studies that were published in a non-indexed journal or in the gray literature, present publication bias. It is common to see that when studies report statistically significant findings they are more likely to be accepted for publication than those that report statistically insignificant findings. For this reason, it is necessary to use rigorous research techniques to identify all possible studies, so as not to fall into the problem of publication bias.^13^

Small studies that present conflicts of interest due to commercial funding, as in the case of studies sponsored by the pharmaceutical industry, may be suspected of publication bias. This is because it is a common practice for studies that reveal negative results for the industry to be withheld from publication. In addition, evaluators should also be suspicious of studies with small sample sizes that show uniformly positive results. These are just some of the indications of possible publication bias.^13^

Thus, when publication bias is suspected, the quality of the evidence should be downgraded by one level, or by two levels when the suspicion is robust. However, when the possibility of publication bias is unlikely, as in the case of funnel plot symmetry, or when the evaluator perceives that the systematic review involved an extensive search of the literature for unpublished studies, there is no need to downgrade the quality of the evidence.^3^

As an example, imagine a systematic review composed of 60 randomized clinical trials seeking to estimate the effects of a new insulin for treating diabetes. Among the studies included, 32 showed a positive effect estimate and all were published. Among the other 28 studies, which were seen as “negative”, only 10 were published. A publication bias of this magnitude can significantly compromise estimates of the effect of bias, which is why the quality of evidence should be downgraded.^3^

The checklist’s analysis of publication bias addresses questions about the breadth of the search strategy and the databases used, about language restrictions, about investigating whether the studies included were influenced by the industry, about funnel plot asymmetry and also about the possibility of discrepancies between published and unpublished trials.^4^

Thus, the objective is to investigate possible factors that could generate some type of publication bias. To assist in this analysis, the GRADE checklist proposes analysis on six questions:

1. Has a broad search been performed?
2. Was the gray literature sought out?
3. Were restrictions on study selection applied, based on language?
4. Was there any industry influence on the studies included in the review?
5. Is there any evidence of asymmetry in funnel plots?
6. Are there any discrepancies between published and unpublished findings among the studies?

The answers to these questions can also vary between the following: 1 = yes; 0 = uncertain; -1 = no; NA1 = not evaluated; or NA2 = not applicable. Through this, upon completion of the review, the assessor will be able to arrive at a transparent judgment of the risk of publication bias through the GRADE tool.^4^

Although not included in the checklist, it is important to mention that another three factors are present in the GRADE system. These need to be evaluated with regard to the possibility of upgrading the level of evidence in observational studies. They comprise large magnitude of effect, presence of a dose-response gradient and residual confounders that increase confidence in the estimate. These factors were not analyzed through the tool, as it was designed specifically for systematic reviews of randomized clinical trials, and these three domains are recommended only in the context of systematic reviews of observational studies.^4^

Next, the factors that increase the quality of evidence in observational studies will be briefly analyzed since, as previously explained, the quality of evidence in these studies starts as low.

**F – Magnitude of effect**

It is important to emphasize that a decision to upgrade the quality of evidence should only rarely be taken if serious limitations are present in any of the five domains previously analyzed.^3^

That said, the next issue to be analyzed is situations of a large magnitude of effect. This may be responsible for raising the level of evidence by one or even two levels, whenever the magnitude of the effect identified in observational studies is large, especially if this effect is observed over a short period.^3^

This is because some interventions made by healthcare professionals present significant reliability, such as antihypertensive drugs for treating high blood pressure, with great confidence in their effectiveness. Thus, in cases in which there is a drastic reduction in the incidence of an outcome after the intervention, the level of evidence should be considered high, even if the evidence comes from observational studies.^6^

From this perspective, when the evidence from observational studies is not downgraded by any of the five domains described above and, in addition, the estimated magnitude of the intervention effect is large or very large, confidence in these results is considerable, thus raising the quality of the evidence. Therefore, it is important to note whether the effect is rapid and consistent, whether the previous disease course is actually reversed by the intervention, and whether the large magnitude of an effect is supported by indirect evidence. Given these analyses, it is possible to make an accurate judgment about this domain.^3^

**G – Dose-response gradient**

The presence of a dose-response gradient is a factor that indicates the occurrence of a cause-effect relationship, which is why it is considered to be another domain that increases the quality of evidence in observational studies.^3^

Thus, if the evaluator identifies a consistent increase in effect from increased exposure, such as the observation that patients receiving anticoagulant treatment with warfarin have a high dose-response gradient due to an increased risk of bleeding, it becomes possible to upgrade the quality of evidence attributable through the presence of a dose-response gradient.^3^

**H – Residual confounders that increase confidence in the estimate**

In observational studies, the quality of evidence starts as low. This is because in these studies there is a high possibility that unmeasured or unknown factors are not adequately balanced between the different groups evaluated, unlike when randomization is used in randomized clinical trials. This is characterized as residual confounding, with overestimation of the effect estimate.^3^

Consequently, in observational studies that have strong methodological rigor, factors associated with the outcomes of interest will be accurately account for, and analyses adjusting for differences in these factors between the intervention and control groups will be conducted. In such cases, the quality can be upgraded, given the evidence for the existence of residual confounders, thereby increasing the level of confidence in the estimate.^6^

As an example, imagine that a rigorous systematic review of observational studies was designed to assess the recovery rate among 30 million sick patients and that the number of patients in non-profit private hospitals was found to be greater than the number in for-profit private hospitals. The factors associated with the outcomes of interest were carefully analyzed and biases relating to the severity of diseases in patients in both types of hospitals were considered. From this, it was possible to upgrade the quality of the initially graded evidence, in view of the quality of the results found.^2^

The GRADE system is a comprehensive and transparent tool for evaluating the evidence and strength of recommendations, and therefore it is very useful in analyzing systematic reviews and developing healthcare guidelines.^3^

## DISCUSSION

The concept of evidence-based healthcare has been used by many professionals. Over the years, numerous systems have emerged as alternatives for classifying and grading the quality of published evidence. The GRADE Working Group is a collaborative working group that aims to minimize the deficiencies of existing classification systems within healthcare, and thus to develop a standardized system for classifying evidence in a transparent and sensible way.^5^

GRADE is already used in more than 110 organizations in 19 countries. Among these, the World Health Organization and the Cochrane Collaboration can be mentioned.^1^ The strength of the study recommendation refers to the possibility that the object of analysis might be adopted or rejected, and this is normally carried out in the analysis of clinical guidelines and technical documents.^3^

It can be seen, then, that judgments about the strength of a recommendation go beyond the analysis of the quality of the evidence. Recommendations can be classified as strong or weak, and this is determined by weighing up the relationship between the advantages (such as improvement in quality of life or increased survival) and the disadvantages (including adverse effects, psychological burden or increased costs).^3^

## CONCLUSION

The translation of this checklist into Portuguese, as reported in this article, provides another tool for assisting in understanding and making better use of the GRADE system. It therefore provides an additional resource for evaluators who do not have much experience with the tool, or who have insufficient time or limited resources to carry out the assessment.

Lastly, it should be noted that GRADE is constantly evolving, due to advances in and development of its approach, with the aim of expanding its use in other areas, such as diagnostic accuracy, economic evaluation and prognosis. These expansions in its use certainly shows that GRADE has fundamental importance and, furthermore, extend assessment of healthcare technologies.^3^
